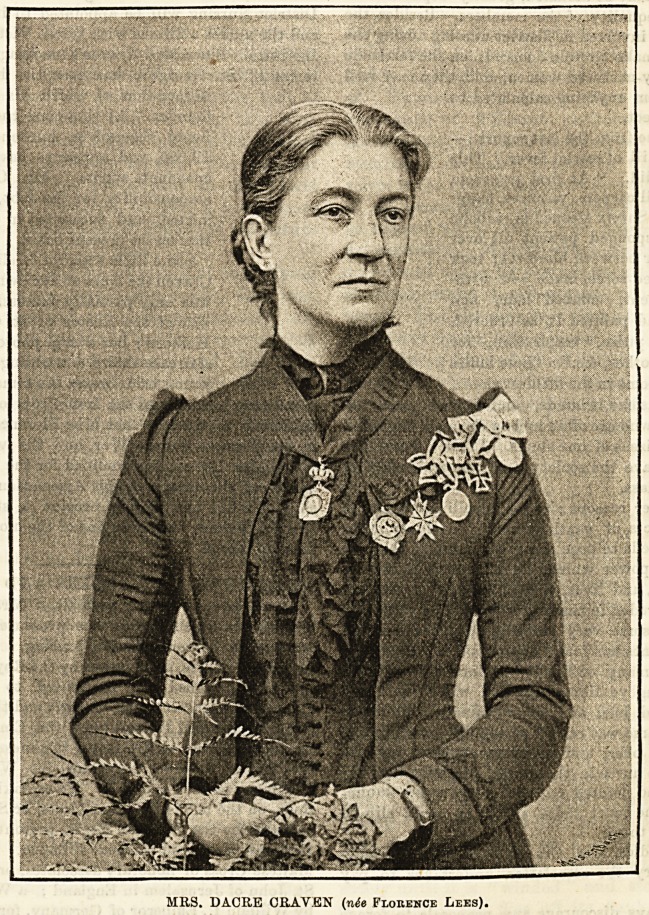# An Interview with Mrs. Dacre Craven (With Portrait)

**Published:** 1890-08-23

**Authors:** 


### An Interview with Mrs. Dacre Craven.

"I want you to be good enough to tell me a little about the nursing organization of which you, I believe, are honorary inspector, Mrs. Dacre Craven."

"I shall be happy to give you any information," is Mrs. Craven's reply as she requests her visitor to be seated in the drawing-room of St. George the Martyr Rectory. "What can I tell you about it?"

"Well, in the first place, please tell me how it originated. I am told that you were the founder of it."

MRS. DACRE CRAVEN (*née* Florence Lees).

"It is even more essential because they haven't the same advantages in their homes and cannot command the same medical attention. I reported to the committee that it seemed to me the system adopted was inefficient, and was connected with an amount of out-door relief, administered very much at the discretion of the nurses, which not only withdrew them from their proper work, but tended very seriously to pauperize their patients."

"And you were not prepared to recommend any extension of that system?"

"No; there was at the time a great outcry about the lack of employment for ladies, and it seemed to me that here was a very suitable sphere for them. I proposed that, not only for superintendents and other officers of the work, but for the nurses also, we should seek among the ranks of educated gentlewomen."

"And you had no doubt as to the practicability of it?"

"I felt sure the idea could be carried out, but there were many who doubted it. Even Miss Florence Nightingale feared its failure, 'but,' she said, 'try it for a year.' I don't know what your politics may be," continued Mrs. Dacre Craven, smilingly—"whether you are a believer in Mr. Gladstone, but he was among the very few who gave me encouragement. 'On the subject of another institution for providing nurses for the poor being really required,' said Mr. Gladstone, 'I won't venture to express any opinion, but I feel great confidence in saying that if you can open a new field for the useful employment of ladies, I am sure you will be doing one of the greatest works of the century.'"

"And eventually your idea was adopted, and a scheme set on foot under your own superintendence?"

"In 1875 the Metropolitan and National Nursing Association was set on foot, and I was its first superintendent."

"Then, as I understand, you receive only ladies of education and some social position, train them as skilled nurses, and employ them in nursing the sick poor in their own homes?"

"Yes; that is the main object of the association, and that we owe entirely to the Duke of Westminster, who was kind enough to use his influence as Chairman of the Committee, that the plan I suggested of having ladies only should have a fair trial. Another object is the establishment of district organizations in various parts of London and throughout the country. It is our aim, also, to raise the standard of nursing, and to improve the social position of nurses."

"Your nurses are paid, of course?"

"Yes; when fully qualified they get £35 a-year for the first year, £38 for the second, and so on increasing £3 every year up to £50, beyond which there is no increase."

"And are they all 'real ladies'?"

"Yes; we take only those who are ladies by birth as well as education."

Of course opinions may differ as to this exclusiveness, but as one of the main objects of the association appears to be to allure into the profession those who can not only bring education and refinement to the work, but can give prestige and *éclat* to this lowly ministry, it may very likely be a point of worldly wisdom to insist on gentle birth in candidates. These ladies live together—half-a-dozen of them, perhaps—in houses the general tone of which it is desirable to maintain at the level of the society to which they have been accustomed, and the harmony and good feeling of which are, it may be assumed, greatly dependent on something like social equality of the members. Besides, the daily routine of work involved in district nursing among the poor must, at times, make terrible demands on the fortitude and nerve of delicately-nurtured women, and they may well have conceded to them anything calculated to sustain their self-respect.

Take a specimen case from the last report:—S. B., a lad of fifteen, ill of scarlet fever. This is what the nurse did. "At first put room into nursing order; all carpets, valences, hangings, &c., removed. Sheets steeped in carbolic hung before door; sponged patient all over with Condy and water between blankets; took precautions against bed-sores, made bed without removal of patient, combed hair, and mopped out throat and painted it, as ordered. Took temperature, pulse, respiration, for doctor. Disinfected clothes, &c." These ladies clean up the foulest rooms in the filthiest slums, dress the most loathsome wounds, and attend most assiduously on patients who are often among the most degraded and vicious of the denizens of our slums. In such work it is a help to them to have the society and companionship of women of their own class.

"And there are other reasons also for enrolling only those who have the instincts of gentlewomen," continues Mrs. Craven, and she proceeds to urge the great value of that indefinable, inscrutable power which real refinement is able to exert on the manners, and lives, and characters of the extremely poor and degraded in times of sickness and suffering. Mrs. Craven relates some very amusing instances in which the gratitude of patients has found expression in almost the only way known to many of them. "Sure now and I'll thrate ye to anything ye like; bedad, I will!" said one native of the Emerald Isle, and she seemingly couldn't be convinced that the nurse was really personally unwilling to accept so liberal an offer, and so when another day the woman met the same nurse in the street, she very heartily renewed her offer, and pointed out that there was a public-house door just up the court there, and they could slip in without a soul seeing them.

"Some of these ladies, Mrs. Craven, are probably independent of the salary the association undertakes to pay them?"

"Yes; but we always discourage any proposals to forego pay. Our desire is that all our nurses and superintendents should be upon the same footing, and this wouldn't be the case if some were paid and others worked gratuitously. We require them, therefore, to accept payment, whether they need it or not."

"You have a central home, I believe, and a number of branches?"

"Yes; in our Central Home in Bloomsbury Square we usually have about nine or ten nurses, and there is a constant succession passing out to supply vacancies in branch homes. Holloway and Paddington were our first branches, and we have also homes at Battersea, Hampstead, Kensington, and Chelsea. In addition to these we have nurses at Westminster, Walworth, Windsor, Bishop Auckland, Hertford, Hereford, and Banbury."

"Then your experiment, commenced in 1874, has really proved a permanent success?"

"Yes, undoubtedly; and we have had the recognition of the Queen Victoria Jubilee Nurses' Institute, and, indeed, have been practically amalgamated with that Institute; their nurses come to us for training in district work."

Mrs. Craven is not now connected with the Metropolitan and National Nursing Association. She has been elected a member of the Council of the Queen Victoria Jubilee Nursing Institute, and for the Nursing Association a paid Inspector of District Nursing will be appointed to inspect all the homes and the nurses affiliated with Queen Victoria Jubilee Nursing Institute. Since Mrs. Craven's marriage, her husband, the rector of St. George's, has been the hon. secretary of the Association of which his wife had been the founder. At that time the existing committee found themselves burdened with a debt of £2,000, and appear to have been in rather despondent spirits. Mr. Craven assumed the responsibility for the debt, formed a new committee, and succeeded in placing the association on its present basis.

Some little attempt to elicit from Mrs. Dacre Craven the facts of her own singular experience was only partially successful. She is now the wife of the Rector of St. George the Martyr, Holborn; but a large photograph on the rectory staircase shows a wedding party in which the young bride wears the remarkable decoration of a military [medal and the Iron Cross of Germany, bearing testimony to the fact that Miss Florence Lees was under fire in the Franco-German War, and the whole experience by which Mrs. Craven has qualified for the post of honorary inspector of the nursing of this Association, and for the authorship of what has already become a standard "Guide to District Nurses," has been one of the most remarkable of the kind on record.

DESCRIPTION OF ORDERS AND DECORATIONS.

As will be seen in our portrait of Mrs. Dacre Craven, she wears a number of decorations presented to her for special services in connection with nursing. Round her neck is suspended a cross, surmounted by the Royal Crown of Prussia, specially designed and presented to her by the Crown Princess of Germany and Prussia and the Princess Royal of Great Britain and Ireland (now the Empress Frederick), for services rendered as Her Royal Highness's superintendent of the Royal Reserve Lazaretto for Wounded Soldiers, at Homburg, in 1870 and 1871. Then, taking the other decorations, on the left is the Red Cross, or Sanitäts Kreutz, surmounted by the Imperial Eagle, presented by Her Imperial Highness the Grand Duchess of Baden. Next come the Silver Maltese Cross of honorary associates, presented by the Order St. John of Jerusalem in England; a War Medal, presented by William I., Emperor of Germany, for services in the field hospitals with the 10th Army Corps in 1870; the Iron Cross, the Order of Merit for Women, presented by William I., Emperor of Germany, for distinguished services in time of war; and, on the right, Queen Victoria's Jubilee Medal, presented by Her Majesty for services rendered to the cause of nursing the sick poor in England.

The heartlessness of the English is exemplified in the following story—told by a Frenchman. After a horrible railway accident an Englishman was heard to remark, "What a nuisance! I can only find half my valet, and my keys are in the pocket of the other half!"

## Figures and Tables

**Figure f1:**